# Hair as an Indicator of Prolonged Paraben Exposure and Its Relation to Weight Gain in a Sample of Spanish Children: A Proof-of-Concept Study

**DOI:** 10.3390/nu17091593

**Published:** 2025-05-06

**Authors:** Yolanda Gálvez-Ontiveros, Patricia González-Palacios, Viviana Ramírez, Celia Monteagudo, Cristina Samaniego-Sánchez, Ana Rivas, Alberto Zafra-Gómez

**Affiliations:** 1 Department of Nutrition and Food Science, University of Granada, 18071 Granada, Spain; yolandagalvez@ugr.es (Y.G.-O.); vivianarl@ugr.es (V.R.); csama@ugr.es (C.S.-S.); amrivas@ugr.es (A.R.); 2 “José Mataix Verdú” Institute of Nutrition and Food Technology (INYTA), Biomedical Research Centre (CIBM), University of Granada, 18071 Granada, Spain; 3 Instituto de Investigación Biosanitaria ibs. GRANADA,18012 Granada, Spain; 4 GENYO, Centre for Genomics and Oncological Research: Pfizer/University of Granada/Andalusian Regional Government PTS Granada, Avenida de la Ilustración, 114, 18016 Granada, Spain; 5 Department of Analytical Chemistry, University of Granada, 18016 Granada, Spain; azafra@ugr.es

**Keywords:** parabens, obesogens, overweight, obesity, schoolchildren, urine, hair, UHPLC-MS/MS

## Abstract

Background: Childhood obesity has become a major public health concern worldwide, and increasing attention is being paid to the potential role of endocrine-disrupting chemicals such as parabens. Understanding environmental contributors is essential for early prevention strategies. Objectives: The aim of the present research was to determine the presence of parabens in hair samples and to examine its association with excess weight and obesity in a sample of Spanish schoolchildren. Methods: A total of 104 cases and 166 controls (3–12 year olds) were recruited. Sociodemographic and lifestyle data and hair and urine samples were gathered. UHPLC-MS/MS coupled to a triple quadrupole detector was used for the quantitative determination of six parabens (methylparaben [MetPB], ethylparaben [EthPB], butylparaben [ButPB], propylparaben [PropPB], and isopropylparaben [i-PropPB]). The relationship between the concentration of parabens in hair and urine was examined according to Spearman correlation coefficients. Finally, binary logistic regression models were constructed to evaluate the relationship of parabens with excess weight/obesity. Results: Detected paraben levels were higher in cases. A weak correlation was produced between hair and urine concentrations, with the exception of i-PropP (hair)/PropPB (urine) and i-PropP (hair)/i-PropPB (urine) in boys, and i-PropPB (hair)/PropPB (urine) in girls. A high level of PropPB was associated with a 4.67 times greater risk of excess weight/obesity only in the boys. Conclusions: In males, a high concentration of PropPB in hair is associated with excess weight and obesity.

## 1. Introduction

Endocrine-disrupting chemicals (EDCs) are exogenous substances that can interfere with the hormonal systems in animals and humans. EDCs can disrupt normal endocrine functioning, which affects hormone synthesis and metabolism, among other aspects. As a result, EDCs may cause adverse health effects culminating in reproductive, developmental, neurological, and immunological issues [1].

Parabens are commonly used preservatives in cosmetic (Regulation (EC) No. 1223/2009), pharmaceutical and food products (Regulation (EC) No. 1333/2008) due to their antimicrobial activity and low allergenic potential [2,3]. Different types of parabens and their permitted concentrations vary depending on the category of product in which they are used, as defined by the corresponding regulatory frameworks. The most commonly used parabens include methylparaben (MetPB), ethylparaben (EthPB), propylparaben (PropPB), and butylparaben (ButPB). The estrogenic activity of parabens increases as a function of alkyl chain length and branching (ButPB > PropPB > MetPB) [4]. Whilst MetPB is the least estrogenic of the aforementioned parabens, it penetrates through the skin at a faster rate and can accumulate in the various layers of the skin [4,5]. After systemic absorption, parabens are able to interact with oestrogen receptors, acting as endocrine disruptors by modulating gene expression and affecting hormone-sensitive tissues [5]. The main sources of exposure to these compounds are diet, personal care products, and environment [3].

In recent decades, exposure to these EDCs has been associated with obesity, given that bisphenols and parabens have a well-known obesogenic capacity. These compounds may alter lipid metabolism and energy balance, which contributes to the development of obesity [6,7]. Evidence for this effect comes from in vitro, in vivo, and epidemiological studies. In vitro studies have shown that parabens can induce the differentiation of pre-adipocyte cells into adipocytes [8,9,10], while in vivo studies have shown that exposure to these compounds is associated with increased body weight and fat accumulation in zebrafish and rodents [11,12,13,14]. Epidemiological studies have linked the presence of parabens in biological samples to excess weight/obesity in humans [15,16]. In addition, the effects of obesogens have been shown to be more potent, even exerting effects at lower concentrations, in early developmental stages, such as infancy, due to the immaturity of the body’s protective systems [6,7].

A variety of biological samples, such as blood and urine, amongst others, have historically been used to determine EDCs exposure [17,18]. However, EDCs analyses in the biological hair matrix has grown in popularity due to a number of advantages over a urine analysis [17,19]. In recent years, however, hair has emerged as a valuable non-invasive biomonitoring matrix, offering several advantages, such as extended detection windows, ease of collection and storage, and a lower risk of contamination during sampling and handling [17,19]. These characteristics make hair particularly suitable for assessing chronic or cumulative exposure to EDCs, especially in populations where conventional sampling may be less feasible. Firstly, unlike urine, which reflects short-term exposure, hair can provide a measure of long-term exposure [17]. In addition, the process is non-invasive with hair samples also being easy to store, transport, and handle.

Based on that discussed above, it is proposed that chronic exposure to parabens in early life, measured through hair analysis, may be associated with an increased risk of excess weight and obesity during childhood. The urine matrix is currently the most commonly used to examine this association. However, given that this reflects only short-term exposure and obesity is a chronic disease, the new approach proposed in the present study is justified by the need for more accurate and less invasive monitoring methods with hair being an ideal biological matrix for revealing long-term EDCs exposure. In this context, the present work aims to study the influence of the presence of parabens in hair (long-term exposure) on excess weight and obesity in a child population.

## 2. Materials and Methods

### 2.1. Study Design and Population

A case-control study was conducted in several health and educational centres in the province of Granada, Spain. Participants were recruited over a two-year period between 2020 and 2022.The inclusion criteria were as follows: (i) a diagnosis of overweight or obesity (in the case of cases only); (ii) prepubertal children aged between 3 and 12 years; and (iii) permanent residence in the study area for at least six months prior to study start. The exclusion criteria were as follows: obesity determined as a symptom of another pathology or as a side effect of pharmacological treatment.

The present study protocol was approved by the ethical committees of the University of Granada and the Provincial Centre for Biomedical Research of Granada (CEI). Parents or legal guardians of the potential participants were informed about the study and signed an informed consent form agreeing to their children’s participation.

**Figure 1:**
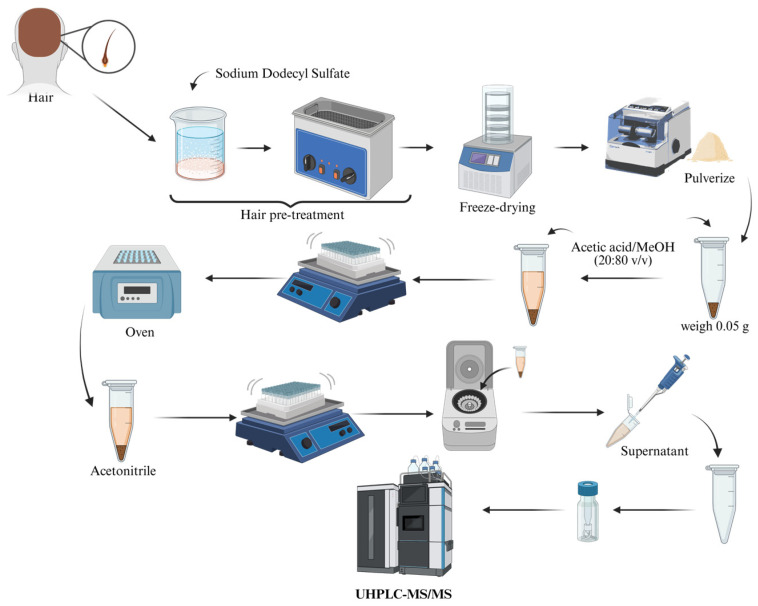
Paraben extraction protocol in hair based on the optimized method by [19].

A total of 194 controls and 128 cases were recruited, resulting in a total of 322 participants. However, there were losses of data and/or samples in the recruitment process, so the final result was a total of 270 participants were included in the investigation (166 controls and 104 cases) (Figure 1).

### 2.2. Data Collection

Trained interviewers conducted face-to-face interviews with parents or legal guardians. Through these interviews, sociodemographic (gender and age of children) and lifestyle data (smoking habits of household members, extracurricular physical activity engagement, and diet) were gathered.

In addition, anthropometric measurements, such as height (cm) and weight (kg), were taken by qualified personnel. The study participants were weighed in light clothing and barefoot using a Tanita portable scale (Tanita Europe B.V. model MC 780-S MA, Amsterdam, The Netherlands). Height was measured using a stadiometer (Seca GmbH & Co. KG. model SECA 214, range 20–207 cm, Hamburg, Germany). When taking height measurements, checks were made to ensure that participants’ backs, buttocks, and heels were in constant contact with the wall. Weight and height were used to calculate body mass index (BMI), which was determined as weight divided by height squared. Participants were then classified as underweight, normal weight, overweight, or obese, according to criteria outlined by Cole et al. (2000, 2007) [20,21].

### 2.3. Determination of Parabens in Biological Samples

As mentioned above, the biological samples collected consisted of hair and urine. Hair samples were obtained on the same day that interviews were conducted, cutting as close as possible to the root in the occipital area of the scalp, as this is the most vascularised area where a higher concentration of toxicants is eliminated. Urine was collected in a polypropylene bottle, striving to collect the first urine of the day due to its higher concentration. Following collection, urine samples were stored at −80 °C whilst awaiting laboratory analysis, while hair samples were kept at room temperature.

A total of six parabens (MetPB, EthPB, PropPB, isopropylparaben [i-PropPB], ButPB, and isobutylparaben [i-ButPB]) were analysed in both hair and urine. The analytical methods used for the extraction and determination of bisphenols and parabens in the selected samples have been previously published in scientific outputs produced by the present research group [19,22]. Nonetheless, these methodologies are briefly described below.

#### 2.3.1. Determination of Parabens in Hair Samples

For the extraction and determination of parabens in hair, the protocol described by Rodríguez-Gómez et al. (2017) [19] (Figure 1) was followed. Hair samples were collected during the initial in-person visit by cutting strands from the posterior vertex region of the scalp. Once in the laboratory, hair was washed following the protocol outlined in the aforementioned work. Next, hair was cut, and 0.05 g of the sample was weighed and incubated with 0.5 mL of an acetic acid/MeOH mixture (20:80, *v*/*v*) at 38 °C for 12 h. After cooling to room temperature, 1 mL of acetonitrile was added for the extraction of analytes. The mixture was vortexed for 15 min and centrifuged for 5 min at 13,000 rpm. The organic phase containing analytes was recovered and evaporated to dryness under a N_2_ stream. The residue was reconstituted using 250 μL of the initial mobile phase. Finally, this was centrifuged at 13,000 rpm and analysed by ultra high-performance liquid chromatography-tandem mass spectrometry (UHPLC-MS/MS(QqQ)) [19]. Data processing and quantification were conducted with the help of MassLynx™ software version 4 (Waters Corporation, Milford, MA, USA).

#### 2.3.2. Determination of Parabens in Urine Samples

**Figure 2:**
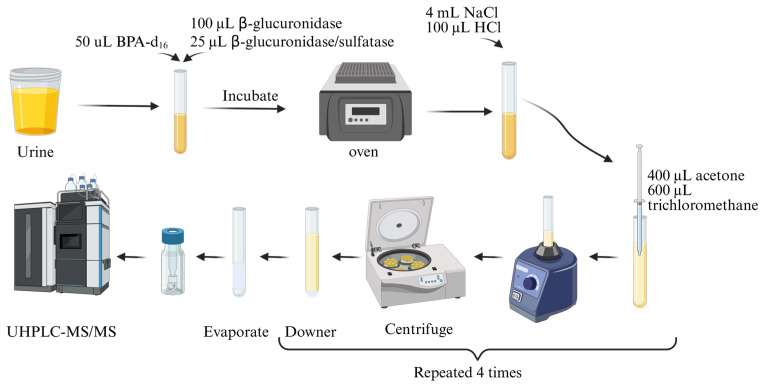
Paraben extraction protocol in urine based on the optimized method by [22].

The method outlined by Moscoso-Ruiz et al. (2022) [22] (Figure 2) was employed for the extraction and determination of the compounds of interest in urine. In summary, β-glucuronidase and β-glucuronidase/arylsulphatase enzymes were added to 4 mL of the sample. Next, 4 mL of 10% (*w*/*v*) sodium chloride (NaCl) and 100 µL of hydrochloric acid (HCl) (6 M) were added. At this point, a dispersive liquid–liquid microextraction was performed by adding 1 mL of the extraction solvents (60% chloroform and 40% *v*/*v* acetone). The mixture was shaken gently and centrifuged for five minutes at 4000 rpm. The sedimented phase was recovered and transferred to a glass tube. This extraction process was repeated three more times, with the total final organic phase then being evaporated to dryness. The dried residue was reconstituted with 100 μL of the initial mobile phase. Finally, this was centrifuged at 13,000 rpm for five minutes and analysed using UHPLC-MS/MS (QqQ) [22]. MassLynx™ software (Waters Corporation) was used for data handling and quantification.

In addition, creatinine levels were determined in the urine samples. This analysis was performed by the Ángel Méndez Soto Clinical Analysis Laboratory. Estimations were determined using the classical Jaffe method, which is based on photometric measurement of creatine reaction kinetics when combined with picric acid at 37 °C [23,24]. The reagent kit was purchased from Biosystems (Barcelona, Spain). The urinary concentrations of the parabens were normalized to creatinine levels to correct for urine dilution.

### 2.4. Statistical Analysis

Data distribution pertaining to continuous parametric variables was summarised according to mean and standard deviation (SD), whilst data distribution pertaining to continuous non-parametric variables was summarised according to the median and interquartile range (IQR). Frequency distributions were calculated for categorical variables. The Student *t*-test (for continuous parametric variables), Mann–Whitney U-test (for continuous non-parametric variables) and Pearson’s Chi-square test (categorical variables) were used to assess differences between the cases and the controls for all variables. The relationship between the concentration of parabens in hair (ng g^−1^) and urine (ng mL^−1^) was determined according to Spearman correlation coefficients.

Finally, a logistic regression model was constructed with hair paraben concentration (ng g^−1^) serving as the independent variable and weight status (overweight and obesity) as the dependent variable. Independent variables were dichotomized according to median values (reference category: concentration ≤ median value). In cases in which the percentage of undetected concentrations of an analyte was >30%, the limit of detection ((LOD)/√2) was used as the cut-off value for dichotomization [25,26,27]. Odds ratios (ORs) and 95% confidence intervals (95% CIs) were calculated for both the crude and adjusted models. Sex, age, energy intake, smoking, physical activity, and body fat percentage were included as confounders in the adjusted models [26,27,28,29]. The influence of each confounder was examined separately with those that modified the OR produced by the crude model by 10% being included in the final adjusted model.

All statistical analyses were performed in IBM SPSS (version 28.0, IBM^®^ SPSS^®^ Statistics, Armonik, NY, USA). Statistical significance was set at *p* ≤ 0.050. 

## 3. Results

Table 1 presents data describing the characteristics of the total sample. Significant differences were observed regarding the variables of age, the smoking habits of members of family units, physical activity engagement, and body fat percentage. In cases, it was observed that both smoking scores and body fat percentages were higher than in the controls, whilst the controls reported greater physical activity engagement (control median = 74.1 vs. case median = 53.8; *p* = 0.001). Table 2 presents study sample characteristics stratified according to sex. The nature and strength of the relationships observed in Table 1 were maintained, with the exception of physical activity engagement in girls, which was not found to significantly differ between the cases and controls (*p* = 0.094).

Table 3 and Table 4 presents the paraben concentrations detected in the hair samples in terms of both the general sample and as a function of sex. Only the concentrations of analytes detected in the present matrix (concentration > LOD) are shown. None of the outcomes presented here were found to be statistically significant either in the overall sample or the sex-stratified samples. In terms of the parabens presented in Table 4, most were detected in higher concentrations in the cases than in the controls. However, the detected concentrations of PropPB in girls and i-PropPB in both boys and girls were higher in the control group.

The correlations produced between the paraben concentrations determined in hair and urine, both for the overall population and as a function of sex, are shown below (Table 5). Statistically significant and direct correlations were identified between the paraben concentrations detected in both biological matrices. These correlations were observed for i-PropPB in the overall sample in boys and girls and for total parabens in girls. For most outcomes, correlation coefficients were lower than 0.600, which suggests a weak relationship between the variables with significant correlations. The only exception to this pertains to i-PropPB in boys, which produced a coefficient of 0.652 (*p* < 0.001) and, therefore, indicates a moderate relationship between the presence of this compound in hair and in urine. The urine concentrations used to establish these correlations are presented in Supplementary Tables S1 and S2.

Table 6 and Table 7 presents outcomes regarding the influence of hair paraben concentrations on excess weight and obesity in both the overall sample and as a function of sex. As shown in Table 6, the association between PropPB and overweight/obesity in boys retains statistical significance from the crude model in the adjusted model (OR = 4.67; *p* = 0.039). No other examined analyte was found to produce a statistically significant outcome in either the overall sample or according to sex (Table 6 and Table 7).

## 4. Discussion

The present investigation focused on determining the influence of hair paraben concentration on the risk of being overweight or obese in a Spanish school population. The main finding was that boys with higher PropPB concentrations were 4.67 times more likely to be overweight or obese.

To the best of our knowledge, no previously conducted studies have investigated the influence of hair paraben concentration on excess weight and obesity in children, with the exception of one study that analysed another potentially obesogenic EDC, namely, bisphenol, using hair samples taken from adults and children to examine the relationship with BMI [30].

At present, scientific evidence is scarce, although several studies have investigated the joint influence of parabens on excess weight and obesity throughout childhood by analysing its presence in urine during the prenatal, postnatal, and/or childhood period [31,32,33,34,35]. In the present study, higher hair paraben concentrations tended to be associated with an increased likelihood of overweight/obesity. In contrast to that reported by previous studies, a longitudinal cohort study conducted by Berger et al. (2021) [33] analysed several parabens and other EDCs in maternal urine and found that prenatal exposure correlated with children’s BMI at five years of age. In this study, higher prenatal exposure to PropPB was found to be associated with a greater likelihood of becoming overweight or obese during early childhood [33].

Another study, which also measured paraben exposure during the prenatal period through maternal urine, found that both the concentration of detected parabens (MetPB, EthPB, PropPB, and ButPB) and the overall concentration tended to be positively associated with a higher weight [31]. Ouidir et al. (2024) [35] conducted a longitudinal study and found an association between postnatal exposure to a mixture of EDCs (phenols and parabens) and a higher BMI at three years of age.

On the other hand, a study conducted in India on 49 obese and 27 non-obese children (aged between 2 and 14 years) found no statistically significant associations between childhood obesity and urinary paraben concentration [32]. Another study carried out in sample of Canadian children evaluated the potential associations between combinations of various EDCs in order to better understand the combined role of these chemical compounds in childhood weight gain [34]. Dugandzic et al. (2024) [34] reported that exposure to mixed parabens and other EDCs increased the risk of being overweight or obese during childhood by 45%, whilst obesity increased by 109% and abdominal obesity by 82%.

With regards to differential effects as a function of sex, some studies have found that the EDCs considered in the present study do act differently according to sex. In the present study, specifically in males, some statistically significant outcomes were obtained. The most relevant of these indicates that higher concentrations of PropPB are positively associated with a greater likelihood of being overweight or obese. Similarly, several previous studies have also observed isolated sex-specific associations [35,36]. In one such study, it was observed that MetPB exposure measured during the third trimester of pregnancy tended to be negatively associated with BMI at three years of age, with this association being stronger in boys [35]. In the case of girls, no statistically significant outcomes were produced in the present study. Whilst, in the present study, total hair paraben concentration in girls was negatively associated with body weight, another study in adolescents observed a positive association between dietary exposure to total parabens and overweight/obesity in adolescent girls [36].

The findings presented above, both overall and in terms of sex, demonstrate a growing concern for the role of individual parabens and overall paraben exposure when it comes to excess body weight and obesity. The findings underline the importance of examining these EDCs at the prenatal and postnatal stage and throughout childhood, whilst also considering the differential effects as a function of sex. However, it is crucial to highlight that the existing inconsistencies in research linking paraben exposure to excess weight/obesity in children may be due to several factors [37]. These inconsistencies may stem from aspects including (i) study design, case-control study (methodology, sample size, type of biological sample, and follow-up length) differences; (ii) variability in EDCs exposure (source and dosage); (iii) individual susceptibility (genetics and health status); and (iv) differences between examined study populations (sociodemographic and socioeconomic differences). All these factors may contribute to the discrepancies between studies.

With regards to the detection of individual EDCs in the present study, MetPB was the main compound detected in hair (100%, see Supplementary Table S3), with detected concentrations ranging from 866.8 to 4668.0 ng g^−1^. This was followed by EthPB, with detected levels ranging from 30.9 to 299.8 ng g^−1^. These findings concur with those reported in previous studies [38,39]. Similarly, these two studies detected MetPB in 100% of the gathered samples, in concordance with the present findings. However, actual measured concentrations were much higher in the work performed by Martin et al. (2019) [38], ranging between 68.3 and 14,187.3 ng g^−1^, whilst those determined by Robin et al. (2024) [39] were found to be lower (1.0–648.0 ng g^−1^). The high presence of MetPB and other parabens reported in the former study may be explained by the populations higher exposure to personal care, hygiene, and food products, which are widely used due to their antimicrobial activity in both the cosmetic and pharmaceutical industry and the food industry [2,3]. Ingestion through food will be low given that only MetPB and EthPB (E214 and E218) are allowed as food additives, together with their respective salts (E215 and E219) [40].

Higher paraben concentrations were found in the hair samples compared to the urine. This finding was also observed in the studies conducted by Robin et al. (2024) [39] and Fäys et al. (2021) [41]. With regards to the associations between MetPB concentrations in the hair and urine samples, the correlations were observed to be non-significant, as was the case in the aforementioned studies [39,41]. Only weak or non-significant correlations were revealed for most of the analysed compounds, with the exceptions of i-PropPB in hair and urine, and PropPB in urine amongst the boys, where moderate correlations were observed. In contrast, strong correlations were found between i-PropPB in hair and PropPB in urine amongst the girls (Supplementary Table S4). This may be explained by the similar chemical makeup of these compounds. A lack of correlation between the measurements taken from both matrices has also been reported in two previous studies, which assessed exposure to other environmental chemical contaminants (pesticides) through hair and urine samples [42,43]. A possible explanation for this may be due to differences in the time window pertaining to each of the samples [42,43].

Human biomonitoring is an essential tool for assessing exposure to environmental chemical contaminants. Urine, together with blood, represents the main matrix used in human biomonitoring [18]. However, these matrices are not highly suited to assessing long-term EDCs exposure due to their short elimination half-life [18,44,45]. Since many EDCs, especially those involving high daily exposure, can accumulate in the body [29,46], some studies have sought to examine their accumulation in body tissues, such as adipose tissue [46] and, even, organs such as the brain [29]. Accumulation of these bisphenols and parabens may not be directly reflected in urine, given that urine concentrations only reflect that which is eliminated by the body at a given time [32]. In order to minimize this limitation, in cases in which the urine matrix is chosen for analysis, it is advised that urine samples be collected longitudinally, at various times of the day and over an extended period. This would likely provide more accurate exposure estimates. It is important to highlight that, when considering chronic disease, real health impacts are exerted by the accumulation of substances, which can cause long-term negative effects [26,47]. This may require an examination of other biological matrices, such as hair, nails, and specific tissues such as adipose tissue [19,46,48]. In the case of adipose tissue, sample collection is invasive and complex. However, hair and nails offer a promising alternative given the fact that sample collection is non-invasive and painless [19,45,46], which is particularly important when working with child populations. In addition, such samples are easy to store, transport, and handle [19,38,48].

The present study presents several strengths. First, it highlights the use of a previously optimized method for the detection of EDCs in hair [19]. Second, it uses a biological sample that reflects long-term exposure to EDCs, which is crucial for relating exposure to parabens with a chronic disease such as obesity. Finally, it represents the first study to simultaneously determine paraben concentration in hair and examine the relationship with excess weight/obesity in children. However, it is important to recognise that the present study is limited by its cross-sectional design, which prevents any conclusions from being made regarding causality when it comes to the relationship between the concentrations of parabens detected in hair and the probability of being overweight/obese during childhood. In addition, the small sample size may affect the interpretation and extrapolation of the findings.

Thus, future longitudinal studies with larger samples and more robust research designs are essential for assessing the long-term effects of paraben exposure on body composition in schoolchildren. Furthermore, the present research highlights the importance of considering simultaneous exposure to multiple contaminants during early childhood and their potential impact on child health, especially the risk of becoming overweight or obese. Additionally, more research is needed to better understand the underlying biological mechanisms and the complex interactions between various chemical compounds and environmental factors in relation to childhood obesity.

## 5. Limitations

This study forms part of a broader, multidisciplinary research initiative aimed at characterizing exposure and the potential health effects of various families of EDCs, including but not limited to parabens, such as bisphenols, benzophenones, perfluorinated compounds, or heavy metals. The current study represents a specific contribution within this larger framework, concentrating on parabens as one of several priority chemical families being examined.

It is important to note, however, that the present study represents a preliminary phase of a larger, ongoing project. Due to the inherent challenges in recruiting participants for this type of study, the current cohort has been used in a proof-of-concept study in which preliminary findings have been allowed to show a significant contribution to the field and to ensure the robustness and impact of future results as our sample size increases.

## 6. Conclusions

The results of this study demonstrate a significant association between elevated levels of propylparaben in hair and a 4.67 times higher increased overweight/obesity risk in boys, with no comparable findings in girls or for other parabens. These findings suggest a potential sex-specific effect of long-term paraben exposure during early childhood. The present findings highlight the importance of investigating the effects of parabens on the most vulnerable populations and the way in which these endocrine-disrupting chemicals influence weight gain as a function of sex in biological matrices reflecting long-term exposure. In addition, further longitudinal studies are needed to better understand the long-term impact of endocrine-disrupting chemicals exposure in children. This highlights the need to implement public health strategies and policies that decrease endocrine-disrupting chemicals exposure as a means of mitigating potential health risks.

## Figures and Tables

**Table 1:** General characteristics of overall sample (n = 270).

		Controls (n = 166)	Cases (n = 104)	*p*-Value
**Gender (%)**	Male	49.4	48.1	0.833 ^a^
	Female	50.6	51.9	
**Age, years**	Mean	7.2	8.9	**<0.001** ^b^
	SD	2.5	2.5	
**Energy intake, Kcal/day**	Mean	2092.2	2057.4	0.647 ^c^
	SD	514.5	568.1	
**Smoking among family** **members (%)**	No	87.9	56.7	**<0.001** ^a^
	Yes	12.1	43.3	
**Physical activity** **(out-of-school) (%)**	No	25.9	46.2	**0.001** ^a^
	Yes	74.1	53.8	
**Body fat percentage**	Median	20.3	33.6	**<0.001** ^b^
	IQR	17.2–22.7	29.58–38.5	

IQR: interquartile range (25th percentile—75th percentile); SD: standard deviation; ^a^ Chi-square test; ^b^ U Mann–Whitney test; ^c^ Student test; outcomes associated with *p* ≤ 0.05 are highlighted in bold.

**Table 2:** General characteristics in boys and girls.

		Boys (n = 132)			Girls (n = 138)		
		Controls (n = 82)	Cases (n = 50)	*p*-Value	Controls (n = 84)	Cases (n = 54)	*p*-Value
**Age, years**	Mean	7.0	8.9	**<0.001 ^a^**	7.4	8.9	**0.001 ^a^**
	SD	2.5	2.5		2.5	2.6	
**Energy intake, Kcal/day**	Mean	2087.9	2226.1	0.212 ^b^	2096.7	1901.4	0.059 ^b^
	SD	473.1	654.0		557.5	426.7	
**Physical activity** **(out-of-school) (%)**	No	19.5	46.0	**0.001 ^c^**	32.1	46.3	0.094 ^c^
	Yes	80.5	54.0		67.9	53.7	
**Smoking among family members (%)**	No	92.6	50.0	**<0.001 ^c^**	83.3	63.0	**0.007 ^c^**
	Yes	7.4	50.0		16.7	37.0	
**Body fat percentage**	Median	18.1	33.6	**<0.001 ^a^**	21.7	33.7	**<0.001 ^a^**
	IQR	15.6–20.6	29.0–38.1		19.8–23.5	30.4–39.0	

IQR: interquartile range (25th percentile–75th percentile); ^a^ U Mann–Whitney test; ^b^ Student test; ^c^ Chi-square test; outcomes associated with *p* ≤ 0.05 are highlighted in bold.

**Table 3:** Hair paraben concentration (ng g^−1^) in the overall sample (n = 270).

	Controls	Cases	
	Median (IQR)	Median (IQR)	*p*-Value
**Methylparaben**	1411 (873.1–3460.3)	1687.8 (866.8–4668.0)	0.447
**Ethylparaben**	66.1 (30.9–260.5)	82.4 (37.1–299.8)	0.490
**Propylparaben**	52.8 (5.8–259.1)	77.9 (19.6–266.6)	0.341
**Isopropylparaben**	2.6 (0.2–8.3)	2.6 (0.2–11.6)	0.764
**Total Parabens**	1803.3 (1050.4–4016.8)	1971.7 (999.1–6137.4)	0.377

IQR: interquartile range (25th percentile–75th percentile); *p*-values indicate the significance of bisphenol intake differences between cases and controls according to Mann–Whitney U.

**Table 4:** Hair paraben concentration (ng g^−1^) in boys and girls.

	Boys (n = 132)			Girls (n = 138)		
	Controls (n = 82)	Cases (n = 50)		Controls (n = 84)	Cases (n = 54)	
	Median (IQR)	Median (IQR)	*p*	Median (IQR)	Median (IQR)	*p*
**Methylparaben**	1411 (748.1–3891.0)	1707.4 (883.0–4106.5)	0.466	1422.3 (887.0–3279.6)	1462.6 (831.0–5856.0)	0.834
**Ethylparaben**	61.9 (29.6–355.8)	91.1 (36.6–267.7)	0.755	70.4 (32.6–245.5)	81.8 (37.6–368.0)	0.481
**Propylparaben**	33.6 (1.0–277.9)	89.7 (23.6–273.0)	0.119	84.1 (13.4–252.6)	60.8 (18.5–221.0)	0.722
**Isopropylparaben**	2.1 (0.2–7.2)	0.2 (0.2–12.1)	0.866	3.4 (0.2–10.5)	3.3 (0.2–10.8)	0.877
**Total** **parabens**	1672.5 (865.9–4371.0)	1971.7 (1245.0–5239.0)	0.327	1919.5 (1134.0–3996.0)	2081.3 (980.0–7012.0)	0.804

IQR: interquartile range (25th percentile–75th percentile); *p*-values indicate the significance of bisphenol intake differences between cases and controls according to Mann–Whitney U.

**Table 5:** Correlations between hair and urine paraben concentrations in the overall sample and in boys and girls separately.

		MetPB	EthPB	PropPB	i-PropPB	Tot. PBs
**Overall Sample**	**Spearman Coef.**	0.007	0.144	0.016	0.496	0.100
	***p*** **-Value**	0.938	0.102	0.855	**<0.001**	0.256
**Boys**	**Spearman coef.**	−0.144	0.078	−0.113	0.652	−0.104
	***p*** **-Value**	0.263	0.534	0.376	**<0.001**	0.408
**Girls**	**Spearman coef.**	0.166	0.177	0.178	0.326	0.308
	***p*** **-Value**	0.193	0.159	0.160	**0.008**	**0.013**

MetPB, methylparaben; EthPB, ethylparaben; PropPB, propylparaben; i-PropPB, isopropylparaben; Tot. PBs, total parabens. Outcomes associated with *p* ≤ 0.05 are highlighted in bold.

**Table 6:** Influence of high hair paraben concentration on weight status (overweight/obese) in the overall sample (n = 270) (low exposure is the reference category).

		Crude		Adjusted		
	OR	95% CI	*p*	OR	95% CI	*p*
**Methylparaben** (High exposure)	1.36	0.83–2.23	0.218	1.14 ^a^	0.43–3.07	0.791
**Ethylparaben** (High exposure)	1.21	0.74–1.98	0.442	1.11 ^b^	0.66–1.87	0.683
**Propylparaben** (High exposure)	1.36	0.83–2.23	0.218	1.53 ^c^	0.84–2.80	0.164
**Isopropylparaben** (High exposure)	1.00	0.61–1.63	1.000	0.91 ^d^	0.34–2.45	0.852
**Total parabens** (High exposure)	1.21	0.74–1.98	0.442	1.13 ^d^	0.42–3.05	0.807

OR: odds ratio; 95% CI: 95% confidence interval. Low exposure ≤ median; High exposure ≤ median. ^a^ Adjusted for gender, age, energy intake, and body fat percentage. ^b^ Adjusted for gender and age. ^c^ Adjusted for gender, age, and energy intake. ^d^ Adjusted for gender, age, energy intake, smoking among family members, and body fat percentage.

**Table 7:** Influence of high hair paraben concentration on weight status (overweight/obesity) in boys and girls separately (low exposure is the reference category).

	Boys (n = 132)						Girls (n = 138)					
	Crude			Adjusted			Crude			Adjusted		
	OR	95% CI	*p*	OR	95% CI	*p*	OR	95% CI	*p*	OR	95% CI	*p*
**Methylparaben** (High exposure)	1.78	0.86–3.66	0.118	1.86 ^a^	0.76–4.59	0.176	1.07	0.54–2.13	0.832	0.59 ^f^	0.16–2.18	0.433
**Ethylparaben** (High exposure)	1.36	0.67–2.75	0.394	1.60 ^b^	0.32–7.89	0.564	1.08	0.55–2.15	0.819	1.17 ^a^	0.50–2.73	0.711
**Propylparaben** (High exposure)	2.42	1.17–4.99	**0.016**	4.67 ^c^	1.08–20.11	**0.039**	0.79	0.40–1.57	0.500	1.05 ^g^	0.29–3.75	0.943
**Isopropylparaben** (High exposure)	0.87	0.43–1.76	0.691	1.55 ^d^	0.30–7.87	0.600	1.14	0.57–2.26	0.715	0.88 ^e^	0.36–2.17	0.794
**Total parabens** (High exposure)	1.52	0.75–3.10	0.245	1.36 ^e^	0.52–3.56	0.527	0.98	0.49–1.94	0.952	0.79 ^g^	0.21–2.89	0.715

OR: odds ratio; 95% CI: 95% confidence interval; outcomes associated with *p*-values < 0.05 are highlighted in bold. Low exposure ≤ median; High exposure ≥ median. ^a^ Adjusted for age and energy intake. ^b^ Adjusted for age, energy intake, physical activity, and body fat percentage. ^c^ Adjusted for age, smoking among family members, and body fat percentage. ^d^ Adjusted for age, energy intake, smoking among family members, physical activity, and body fat percentage. ^e^ Adjusted for age, energy intake, and smoking among family members. ^f^ Adjusted for age, energy intake, and body fat percentage. ^g^ Adjusted for age, energy intake, smoking among family members, and body fat percentage.

## Data Availability

The original contributions presented in this study are included in the article. Further inquiries can be directed to the corresponding author.

## Supplementary Materials

The following supporting information can be downloaded at: https://www.mdpi.com/article/10.3390/nu17091593/s1, Table S1. Paraben concentration in urine (ng mL^−1^) in the overall sample; Table S2. Paraben concentration in urine (ng mL^−1^) in boys and girls individually; Table S3. Frequency of detection (%) of parabens in hair; Table S4. Correlations between bisphenol and paraben concentration in hair and urine in the overall sample and in boys and girls individually.

## Abbreviations

The following abbreviations are used in this manuscript:

BMI	Body mass index
ButPB	Butylparaben
CEI	Centre for Biomedical Research of Granada
CI	Confidence interval
EDCs	Endocrine-disrupting chemicals
EthPB	Ethylparaben
HCl	Hydrochloric acid
IQR	Interquartile range
i-ButPB	Isobutylparaben
i-PropPB	Isopropylparaben
LOD	Limit of detection
MetPB	Methylparaben
NaCl	Sodium chloride
OR	Odds ratio
PropPB	Propylparaben
SD	Standard deviation
UHPLC-MS/MS	Ultra-high performance liquid chromatography-tandem mass spectrometry
