# Supplementary material for: Hair as an Indicator of Prolonged Paraben Exposure and Its Relation to Weight Gain in a Sample of Spanish Children: A Proof-of-Concept Study
Source: Nutrients. 2025 May 6;17(9):1593. doi: 10.3390/nu17091593 (PMC12073634; doi:10.3390/nu17091593)
Supplement: Supplementary file 1 [file nutrients-17-01593-s001.zip › nutrients-3589115-supplementary.pdf]

nutrients

MDPI

Table S1. Paraben concentration in urine (ng mL^{-1}) in the overall sample.

	Controls	Cases	*p*-value
	Median (IQR)	Median (IQR)	
Methylparaben	3.85	4.70	3.85
Ethylparaben	2.05–8.30	2.34–21.48	2.05–8.30
Propylparaben	2.34–21.48	0.02	2.34–21.48
Isopropylparaben	0.02	0.02–0.02	0.02
Total Parabens	0.02–0.13	0.02	0.02–0.13

IQR: interquartile range (25th percentile–75th percentile); *p*-value pertaining to differences in bisphenol and paraben intake between cases and controls according to the Mann–Whitney U.

Table S2. Paraben concentration in urine (ng mL^{-1}) in boys and girls individually.

	Boys (n = 132)			Girls (n = 138)		
	Controls (n = 82)	Cases (n = 50)	*p*-value	Controls (n = 84)	Cases (n = 54)	*p*-value
	Median (IQR)	Median (IQR)		Median (IQR)	Median (IQR)	
Methylparaben	3.50 (2.26–7.24)	4.70 (2.54–20.95)	0.298	4.00 (1.68–10.11)	4.53 (2.23–23.61)	0.801
Ethylparaben	0.02 (0.02–0.11)	0.02 (0.02–0.02)	0.334	0.02 (0.02–0.14)	0.02 (0.02–0.09)	0.809
Propylparaben	0.02 (0.02–0.02)	0.76 (0.02–2.38)	**0.008**	0.02 (0.02–1.60)	0.02 (0.02–1.55)	0.894
Isopropylparaben	0.02 (0.02–0.02)	0.02 (0.02–0.20)	**0.009**	0.02 (0.02–0.20)	0.02 (0.02–0.07)	0.700
Total Parabens	3.81 (1.88–7.29)	4.96 (2.82–19.48)	0.148	4.88 (1.88–11.44)	5.77 (2.59–38.16)	0.197

IQR: interquartile range (25th percentile–75th percentile); *p*-value pertaining to differences in bisphenol and paraben intake between cases and controls according to the Mann–Whitney U.

Table S3. Frequency of detection (%) of parabens in hair.

	Frequency of Detection (%)
Methylparaben	100
Ethylparaben	99
Propylparaben	81
Isopropylparaben	54

Table S4. Correlations between bisphenol and paraben concentration in hair and urine in the overall sample and in boys and girls individually.

			Urine (ng mL⁻¹)				
			**MetPB**	**EthPB**	**PropPB**	**i-PropPB**	**Tot. PBs**
**Hair (ng g⁻¹) in overall sample**	Methylparaben	**Spearman coef.**	0.007	0.062	0.163	0.148	0.106
		***p*-value**	0.938	0.481	0.067	0.091	0.227
	Ethylparaben	**Spearman coef.**	−0.023	0.144	−0.007	0.067	0.048
		***p*-value**	0.799	0.102	0.933	0.450	0.589
	Propylparaben	**Spearman coef.**	0.059	0.134	0.016	−0.011	0.070
		***p*-value**	0.516	0.127	0.855	0.898	0.426
	Isopropylparaben	**Spearman coef.**	0.057	−0.021	0.597	0.496	0.235
		***p*-value**	0.530	0.808	**<0.001**	**<0.001**	**0.007**
	Total parabens	**Spearman coef.**	0.006	0.077	0.123	0.109	0.100
		***p*-value**	0.945	0.384	0.167	0.216	0.256
			**Urine (ng mL⁻¹)**				
			**MetPB**	**EthPB**	**PropPB**	**i-PropPB**	**Tot. PBs**
**Hair (ng g⁻¹) in boys**	Methylparaben	**Spearman coef.**	−0.144	0.173	0.005	0.099	−0.077
		***p*-value**	0.263	0.164	0.972	0.430	0.537
	Ethylparaben	**Spearman coef.**	−0.241	0.078	−0.092	0.127	−0.136
		***p*-value**	0.059	0.534	0.472	0.310	0.277
	Propylparaben	**Spearman coef.**	0.013	0.168	−0.113	0.018	0.008
		***p*-value**	0.920	0.177	0.376	0.885	0.950
	Isopropylparaben	**Spearman coef.**	0.056	−0.075	0.481	0.652	0.194
		***p*-value**	0.666	0.550	**<0.001**	**<0.001**	0.119
	Total parabens	**Spearman coef.**	−0.160	0.162	−0.026	0.075	−0.104
		***p*-value**	0.215	0.195	0.841	0.551	0.408
			**Urine (ng mL⁻¹)**				
			**MetPB**	**EthPB**	**PropPB**	**i-PropPB**	**Tot. PBs**
**Hair (ng g⁻¹) in girls**	Methylparaben	**Spearman coef.**	0.166	−0.025	0.315	0.185	0.300
		***p*-value**	0.193	0.842	**0.011**	0.141	**0.015**
	Ethylparaben	**Spearman coef.**	0.200	0.177	0.112	−0.004	0.245
		***p*-value**	0.115	0.159	0.376	0.975	**0.049**
	Propylparaben	**Spearman coef.**	0.126	0.084	0.178	−0.031	0.135
		***p*-value**	0.326	0.507	0.160	0.805	0.284
	Isopropylparaben	**Spearman coef.**	0.061	0.038	0.715	0.326	0.284
		***p*-value**	0.635	0.764	**<0.001**	**0.008**	**0.022**
	Total parabens	**Spearman coef.**	0.173	0.003	0.270	0.133	0.308
		***p*-value.**	0.174	0.984	**0.031**	0.290	**0.013**

Outcomes associated with $p \leq 0.05$ are highlighted in bold.
